# Supplementary material for: Small body size is associated with increased evolutionary lability of wing skeleton proportions in birds
Source: Nat Commun. 2024 May 28;15:4208. doi: 10.1038/s41467-024-48324-y (PMC11133451; doi:10.1038/s41467-024-48324-y)
Supplement: Supplementary file 2 — Reporting Summary [file 41467_2024_48324_MOESM2_ESM.pdf]

nature portfolio

Corresponding author(s): Andrew Orkney

Last updated by author(s): 2024 03 20

Reporting Summary

Nature Portfolio wishes to improve the reproducibility of the work that we publish. This form provides structure for consistency and transparency in reporting. For further information on Nature Portfolio policies, see our Editorial Policies and the Editorial Policy Checklist.

Statistics

For all statistical analyses, confirm that the following items are present in the figure legend, table legend, main text, or Methods section.

n/a	Confirmed	
☐	☒	The exact sample size (n) for each experimental group/condition, given as a discrete number and unit of measurement
☐	☒	A statement on whether measurements were taken from distinct samples or whether the same sample was measured repeatedly
☐	☒	The statistical test(s) used AND whether they are one- or two-sided<br>*Only common tests should be described solely by name; describe more complex techniques in the Methods section.*
☐	☒	A description of all covariates tested
☐	☒	A description of any assumptions or corrections, such as tests of normality and adjustment for multiple comparisons
☒	☐	A full description of the statistical parameters including central tendency (e.g. means) or other basic estimates (e.g. regression coefficient) AND variation (e.g. standard deviation) or associated estimates of uncertainty (e.g. confidence intervals)
☐	☒	For null hypothesis testing, the test statistic (e.g. F, t, r) with confidence intervals, effect sizes, degrees of freedom and P value noted<br>*Give P values as exact values whenever suitable.*
☒	☐	For Bayesian analysis, information on the choice of priors and Markov chain Monte Carlo settings
☒	☐	For hierarchical and complex designs, identification of the appropriate level for tests and full reporting of outcomes
☐	☒	Estimates of effect sizes (e.g. Cohen's d, Pearson's r), indicating how they were calculated

Our web collection on statistics for biologists contains articles on many of the points above.

Software and code

Policy information about availability of computer code

Data collection	No software was used for data collection, as all data analysed in this study were drawn from public repositories (see Data section).
Data analysis	All analyses were conducted in R version 4.2.3.<br>Custom functions are available in the GitHub repository identified in the manuscript and its associated Zenodo deposition:<br>https://zenodo.org/doi/10.5281/zenodo.10879690<br>Existing functions from the following packages were used in analysis:<br>Geomorph version 4.0.5<br>phytools version 1.5-1<br>nlme version 3.1-162<br>caTools version 1.18.2<br>car version 3.1.1<br>abind version 1.4-5<br>ape version 5.0<br>dplyr version 1.1.1<br>reshape2 version 1.4.4<br>zoo version 1.8-12<br>The following packages were used to produce visualisations:<br>cowplot version 1.1.1<br>dendextend version 1.17.1<br>geomtextpath version 0.1.1

ggdendro version 0.1.23
ggnewscale version 0.4.9
ggplot2 version 3.4.1
ggpubr version 0.6.0
ggrepel version 0.9.3 (used in the Zenodo version of the figures, but labels were placed manually in the final typeset figures)
jpeg version 0.1-10
magick version 2.8.2
png version 0.1-8

For manuscripts utilizing custom algorithms or software that are central to the research but not yet described in published literature, software must be made available to editors and reviewers. We strongly encourage code deposition in a community repository (e.g. GitHub). See the Nature Portfolio guidelines for submitting code & software for further information.

Data

Policy information about availability of data

All manuscripts must include a data availability statement. This statement should provide the following information, where applicable:

- Accession codes, unique identifiers, or web links for publicly available datasets
- A description of any restrictions on data availability
- For clinical datasets or third party data, please ensure that the statement adheres to our policy

Original skeletal landmark constellations were sourced from Navalón et al., 2022: https://doi.org/10.1038/s41586-022-05372-y
The original phylogenies used to represent evolutionary non-independence were sourced from Prum et al. 2015 and Oliveros et al. 2019 (a combined tree is also available from the aforementioned publication, original trees can be sourced from: https://doi.org/10.5281/zenodo.28343 https://doi.org/10.5061/dryad.2vd01gr https://osf.io/wjk3m/).
Figures presented in the paper herein can be generated from the codes available in our GitHub repository: https://github.com/aorkney/TinyBirds
Zenodo deposition: https://zenodo.org/doi/10.5281/zenodo.10879690

Research involving human participants, their data, or biological material

Policy information about studies with human participants or human data. See also policy information about sex, gender (identity/presentation), and sexual orientation and race, ethnicity and racism.

Reporting on sex and gender	N/A
Reporting on race, ethnicity, or other socially relevant groupings	N/A
Population characteristics	N/A
Recruitment	N/A
Ethics oversight	N/A

Note that full information on the approval of the study protocol must also be provided in the manuscript.

Field-specific reporting

Please select the one below that is the best fit for your research. If you are not sure, read the appropriate sections before making your selection.

☐ Life sciences ☐ Behavioural & social sciences ☒ Ecological, evolutionary & environmental sciences

For a reference copy of the document with all sections, see nature.com/documents/nr-reporting-summary-flat.pdf

Ecological, evolutionary & environmental sciences study design

All studies must disclose on these points even when the disclosure is negative.

Study description	This study investigated patterns of evolutionary covariance between skeletal proportions across a representative dataset of 228 bird species in a phylogenetic comparative framework. We determine the role of body mass variety in establishing this structure and show that size scaling is accompanied by a systemic reorganisation of evolutionary modules within the avian skeleton.
Research sample	We use the bird skeletal landmark configurations presented in Navalón et al., 2022, because they range over 3.5 orders of magnitude of body mass variation, and because they are ecologically and phylogenetically diverse.
Sampling strategy	Methods 1 and 2 involve subsampling procedures. A subsample of n=30 was employed for any individual subsampled analysis within method 1, and 30 permutations with resampling were performed in method 1, whereas 100 permutations were used

	in method 2. Re-sampling in method 2 was conducted 100 times, by dividing the range of body masses into 10 equal bins, and subsampling up to 10 species within each bin to produce a subsample with a Gaussian distribution of body mass values. This step is necessary because downstream statistical tests assume that skewness in the body mass distribution can be neglected. Individual subsample sizes were allowed to vary, so that a variety of plausible Gaussian distributions could be explored and to ensure that variations in taxonomic sampling completeness across the avian tree do not confound analyses. An identical subsampling procedure was conducted on a permuted version of the original data, to produce a null distribution of downstream test statistics for comparison.
Data collection	No new data was collected in this study; see Navalón et al., 2022 for a description of original data collection.
Timing and spatial scale	These data do not possess inherent spatio-temporal scales. They represent the anatomical proportions of a diverse cohort of extant birds sourced from all over the world.
Data exclusions	No data excluded.
Reproducibility	The scripts which produce our figures and reported summary statistics were run independently by AO and BPH to verify we reached the same conclusions. These scripts are commented and are available for replication on our dedicated Git repository and Zenodo deposition, and their function was independently tested during peer review.
Randomization	Body mass grouping are described in detail in methods 1 and 2 in text. Method 1, a rolling window of 40 taxa (from the smallest to largest) was employed, and moved incrementally to divide the dataset into 20 overlapping bins.<br>In method 2, up to 10 taxa were sampled randomly between 10 evenly spaced breaks of log10(body mass) across birds, to produce pseudo-Gaussian sampling distributions.
Blinding	Blinding was not performed in this study. The source data were already public, and an interrogation of the data was necessary to design a meaningful analysis- for example, identifying combinations of bones in Method 1 that were subsequently investigated further in Method 2.

Did the study involve field work? ☐ Yes ☒ No

Reporting for specific materials, systems and methods

We require information from authors about some types of materials, experimental systems and methods used in many studies. Here, indicate whether each material, system or method listed is relevant to your study. If you are not sure if a list item applies to your research, read the appropriate section before selecting a response.

Materials & experimental systems

n/a	Involved in the study
☒	☐ Antibodies
☒	☐ Eukaryotic cell lines
☒	☐ Palaeontology and archaeology
☒	☐ Animals and other organisms
☒	☐ Clinical data
☒	☐ Dual use research of concern
☒	☐ Plants

Methods

n/a	Involved in the study
☒	☐ ChIP-seq
☒	☐ Flow cytometry
☒	☐ MRI-based neuroimaging

Plants

Seed stocks	N/A
Novel plant genotypes	N/A
Authentication	N/A
